# Classification of motor imagery tasks for BCI with multiresolution analysis and multiobjective feature selection

**DOI:** 10.1186/s12938-016-0178-x

**Published:** 2016-07-15

**Authors:** Julio Ortega, Javier Asensio-Cubero, John Q. Gan, Andrés Ortiz

**Affiliations:** 1 Department of Computer Architecture and Technology, CITIC, University of Granada, Granada, Spain; 2 Neuralcubes Ltd, London, UK; 3 School of Computer Science and Electronic Engineering, University of Essex, Colchester, UK; 4 Department of Communications Engineering, University of Malaga, Málaga, Spain

**Keywords:** Brain-computer interfaces (BCI), Feature selection, EEG classification, Imagery tasks classification, Multiobjective optimization, Multiresolution analysis (MRA)

## Abstract

**Background:**

Brain-computer interfacing (BCI) applications based on the classification of electroencephalographic (EEG) signals require solving high-dimensional pattern classification problems with such a relatively small number of training patterns that curse of dimensionality problems usually arise. Multiresolution analysis (MRA) has useful properties for signal analysis in both temporal and spectral analysis, and has been broadly used in the BCI field. However, MRA usually increases the dimensionality of the input data. Therefore, some approaches to feature selection or feature dimensionality reduction should be considered for improving the performance of the MRA based BCI.

**Methods:**

This paper investigates feature selection in the MRA-based frameworks for BCI. Several wrapper approaches to evolutionary multiobjective feature selection are proposed with different structures of classifiers. They are evaluated by comparing with baseline methods using sparse representation of features or without feature selection.

**Results and conclusion:**

The statistical analysis, by applying the Kolmogorov-Smirnoff and Kruskal–Wallis tests to the means of the Kappa values evaluated by using the test patterns in each approach, has demonstrated some advantages of the proposed approaches. In comparison with the baseline MRA approach used in previous studies, the proposed evolutionary multiobjective feature selection approaches provide similar or even better classification performances, with significant reduction in the number of features that need to be computed.

## Background

Brain computer interfaces (BCI) try to identify the cognitive states of the user to control a computer or any other kind of devices. These systems promote interesting and useful applications based on this new way of human–machine communication, such as those related with the improvement of quality of life for people with disabilities. Electroencephalography (EEG) is a signal acquisition technique that is widely used for BCI as it is not expensive compared to other methods and does not require surgery to place electrodes. Nevertheless, BCI systems based on the classification of EEG signals pose a high-dimensional pattern classification problem [1], due to (1) the presence of noise or outliers (as EEG signals have a low signal-to-noise ratio); (2) the need to represent time information in the features (as brain signal patterns are related to changes in time); (3) the non-stationarity of EEG signals, which may change quickly over time, subjects, or within experiments. Moreover, the curse of dimensionality is usually present in the classification of EEGs as the number of patterns (EEGs) available for training is relatively small, and the number of features is usually much larger than the number of available training patterns. This way, as many other high-dimensional pattern classification or modeling tasks, BCI requires feature selection techniques in order to remove redundant, noise-dominated, or irrelevant inputs. In particular, dimensionality reduction is very important to improve the accuracy and interpretability of the classifiers when the number of features is too large compared to the number of available training patterns. Thus, by reducing the dimension of the input patterns, it is possible to (1) decrease the computational complexity (2) remove irrelevant/redundant features that would make it more difficult to train the classifier, and (3) avoid the curse of dimensionality [2]. There are two main alternatives for dimensionality reduction: feature extraction, which generates a pattern space of lower dimension by applying a transformation to the patterns to be classified, and feature selection that simply chooses some features from the original set according to some criteria. This paper deals with feature selection because interpretability is important for BCI applications. Feature extraction may be more efficient in dimensionality reduction, but it usually results in a loss of the interpretability of the new feature space.

Among the three main approaches for feature selection: filter, wrapper, and embedded methods [3], our proposals in this paper lie inside the wrapper alternative as wrapper and embedded methods are usually recognized as the preferable approaches whenever they would be feasible [4]. Nevertheless, as the size of the search space depends exponentially on the number of possible features, an exhaustive search for the best feature set is almost impossible when the feature dimension is high. Thus, new metaheuristics, such as evolutionary computation, and parallel processing could be considered as an interesting alternative [5] to take the advantage of high performance computer architectures for feature selection not only by using wrapper, but also for filter methods.

In [6] and [7], multiobjective optimization has been proposed for feature selection, respectively inside wrapper and filter methods, in a multiresolution analysis (MRA) system for BCI [8]. MRA applies a sequence of successive approximation spaces that satisfy a series of constraints to reach a description as close as possible to a target signal [9], and thus it is useful whenever the target signal presents different characteristics in the successive approximation spaces. A specific example of MRA systems, the discrete wavelet transform (DWT), has been applied in [8] to characterize EEGs from motor imagery.

Motor imagery (MI) is a BCI paradigm that uses the series of amplifications and attenuations of short duration occasioned by limb movement imagination, the so-called event related desynchronization (ERD) and event related synchronization (ERS). The task of ERD/ERS analysis is complex because they are weak and noisy and occur at different locations of the cortex, at different instants within a trial, and in different frequency bands. This may lead to high-dimensional patterns making the number of available patterns to conduct ERD/ERS analysis significantly less than the number of features. This paper extends the experiments and comparisons and conclusions given in [6] by providing more details about the characteristics of the cost functions used in the multiobjective optimization procedure and including new methods for feature extraction (such as sparse representations) and classification (such as SR-SVC).

## Methods

### Multiobjective optimization in supervised feature selection

Our multiobjective optimization procedure for feature selection has been implemented through a *wrapper* approach, which can be seen as a search for the feature set that optimizes a cost function that evaluates the utility of the given features according to the performance attained by the classifier. Although the performance of a classifier can be expressed by its accuracy for a given set of patterns, other measures that quantify properties such as the generalization capability and computational efficiency should be taken into account. This way, a multiobjective formulation for the feature selection problem could constitute a powerful approach to feature selection. Thus, whenever the selection of features is optimized for both accuracy and generalization capability by using the training patterns, better accuracies could be provided by the classifier when test (or working) patterns are applied.

A multiobjective optimization problem can be defined as finding a vector of decision variables **x** = [x_1_,x_2_,…,x_n_] ∈ R^n^ that satisfies a restriction set, e.g., g(**x**) ≤ 0, h(**x**) = 0, and optimizes a function vector **f**(**x**), whose scalar values (f_1_(**x**), f_2_(**x**),…, f_m_(**x**)) represent the objectives of the optimization. As these objectives are usually in conflict, instead of providing only one optimal solution, the procedures applied to multiobjective optimization should obtain a set of *non*-*dominated* solutions, known as Pareto optimal solutions, from which a decision agent will choose the most convenient solution in specific circumstances. These Pareto optimal solutions are optimal in the sense that in the corresponding hyper-area known as *Pareto front*, no solution is worse than the others when all the objectives are taken into account.

Feature selection as a multiobjective optimization problem can be for either supervised or unsupervised classifiers. A thorough review on this topic is given by Handl and Knowles [10]. With respect to supervised classifiers, multiobjective feature selection procedures often take into account the number of features and the performance of the classifier [11, 12]. There are a lot of studies focusing on feature selection for unsupervised classification [10, 13, 14]. As the labels for the training and testing patterns are available in our BCI datasets, this paper deals with supervised multiobjective feature selection.

**Fig. 1:**
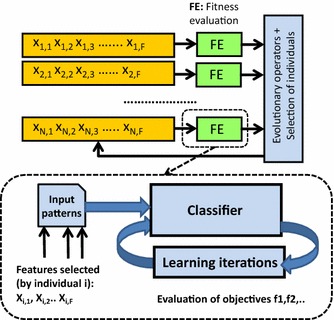
Wrapper approach to feature selection by evolutionary multiobjective optimization

Figure 1 provides a scheme of multiobjective optimization for feature selection in a classification procedure. This scheme corresponds to the wrapper approach for feature selection implemented in our study. Each individual of the population encodes the features of the input patterns that are taken into account during the classifier training. An individual evaluation implies to train the classifier with the given input patterns and to determine the classifier performance by using several cost functions, as a multiobjective optimization procedure has been considered. From Fig. 1 the usefulness of a multiobjective approach for feature selection is apparent as the classifier’s behavior is not usually characterized by only one parameter. Besides the accuracy, there are other measures that quantify its performance. Among them we have measures to evaluate its generalization capabilities or the possible amount of overfitting the classifier could present.

The main steps of the multiobjective evolutionary algorithm correspond to those of NSGA-II (non-dominated Sorting Genetic Algorithm-II) [15], including the specific individual codification and genetic operators implemented for the application at hand (information about them will be provided below in the section devoted to results). In NSGA-II the fitness values of the individuals in the population are sorted according to the different fronts of nondominated individuals (nondomination levels) where they belong, while the diversity among individuals in the same nondominated front is also preserved. To maintain the diversity among solutions with the same nondominance level, NSGA-II estimates the density of solutions surrounding a given solution through the average distance of the nearest neighbour solutions on either side of the considered solution for each dimension (objective) of the front.

In this paper we have used two cost functions that take into account the available knowledge about the classes to which the training patterns belong to. Moreover, to characterize the performance of the classifier while it has been trained or adjusted for a given set of features (an individual of the population), it is important not only to take into account the accuracy obtained for the training set but also to its behavior for unseen instances, i.e., its generalization capabilities. Thus, the first of the two cost functions is related with the Kappa index [16], which provides an accurate description of the classifier performance. This Kappa index can be considered even better than the classification ratio as it takes into account the per class error distribution. The other cost function evaluates aspects such as the generalization capability or the classifier overfitting. In our case, tenfold cross-validation analysis to the training patterns was applied to obtain the cost function values. In the section on experimental results we provide some information to show that both cost functions allow us to implement a tradeoff among the accuracy of the classifier and their generalization capability. A lower value on the first function could also imply overfitting, and thus a higher value in the second cost function.

### Multiresolution analysis of EEG for BCI

The dataset we have used to evaluate the proposed multiobjective feature selection procedures was recorded in the BCI Laboratory at the University of Essex. It includes patterns that correspond to three different classes of imagined movements (right hand, left hand, and feet) from 10 subjects aged from 24 to 50 (58 % female, 50 % naïve to BCI) and was recorded with a sampling frequency of 256 Hz during four different runs. There are a total of 120 trials for each class for each subject. More details about this dataset can be found in [8].

Each pattern was obtained from an EEG trial by the feature extraction procedure based on the MRA described in [8]. Thus, each signal obtained from each electrode contains several segments to which a set of wavelets detail and approximation coefficients are assigned. With respect to the family of wavelets used, in [8] wavelet lifting (also known as second generation wavelets) is considered to build a set of wavelets adequate to cope with the temporal, spectral and spatial domains present in the ERS/ERD analysis. The lifting scheme proposed in [8] is based on a graph representation of a motor imagery trial and builds the applied wavelets family more straightforward and with low resource consumption.

**Fig. 2:**
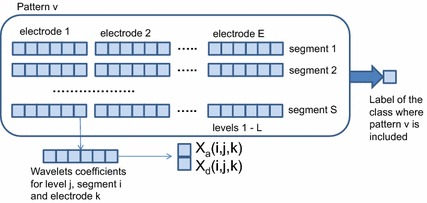
Characterization of an EEG signal (pattern) in [9]

This way, assuming there are S segments, E electrodes, and L levels of wavelets, each pattern is characterized by 2 × S×E × L sets of coefficients (the number of coefficients in each level set depends on the level). In the Essex BCI dataset, S = 20 segments, E = 15 electrodes, and L = 6 levels, therefore there are 3600 sets in total, with from 4 to 128 coefficients in each set to characterize each pattern (a total of 151,200 coefficients). Figure 2 shows how the pattern features are generated. Taking into account that the number of training patterns for each subject is approximately 180, it is clear that an efficient procedure for feature selection is required.

In [8] a simple approach to reduce the number of coefficients is applied, in which only one coefficient is assigned to each electrode and each level of approximation and detail. This coefficient is obtained by computing the second moment of the coefficient distribution (variance) and normalizing the value between 0 and 1. This way, the number of coefficients for a given pattern is 2 × S × E × L.

**Fig. 3:**
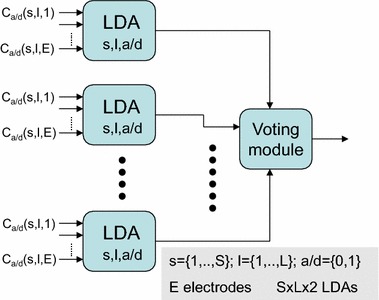
EEG classification with multiple LDA classifiers based on majority voting, with one LDA classifier per segment per wavelet level and per type of coefficient [8]

The approach proposed in [8] to cope with the problem of curse of dimensionality can be understood from Fig. 3, where a set of LDA (linear discriminant analysis) classifiers are used in the first layer. Then, a module for majority voting of all the LDA outputs is used to provide the final classification output. A set of 2 × S × L LDA classifiers with the number of inputs equaling the number of electrodes are adopted, as shown in Fig. 3. Each LDA module receives as many inputs as electrodes. As this number is much lower than the number of training patterns, the curse of dimensionality is avoided. This is the approach we consider as one of the reference or baseline approaches in this paper.

### Sparse representation for feature extraction based on MRA

**Fig. 4:**
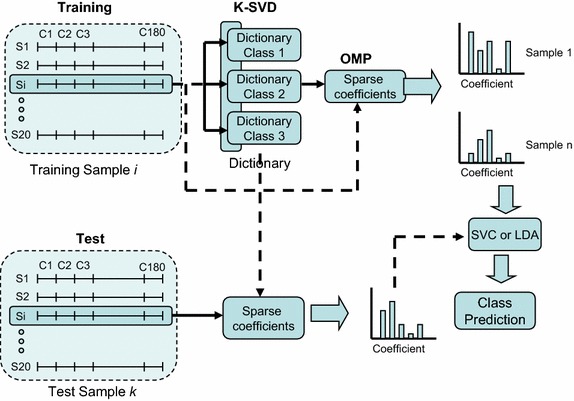
Classification based on a bag of sparse features

It is also possible to reduce the number of MRA features by using sparse representation [17]. This methodology has been applied here to determine the set of features in both LDA and SVC (support vector classifier), as shown in Fig. 4, for comparison with the multiobjective feature selection methods proposed in this paper.

According to the MRA used to describe the EEGs, the coefficients for each EEG segment are grouped for all channels and then used to construct a dictionary. This way each segment has 2xL coefficients (2 × 6 = 12 in this case) from each of E electrodes (15 in this case), which can be arranged in a vector x_i_ ∈ R^2 × L × E^ (12 × 15 = 180 components in this case). These x_i_ vectors, corresponding to the different segments, can be used as training samples to learn a representative dictionary for each class. The goal is to obtain sufficient representative features by a sparse representation [17] that can be built from a linear combination of a small number of elementary signals. These signals, called atoms, are chosen from an over-complete dictionary composed by a number of prototypes that exceeds the dimension of the signal space. This way, a signal y (with 2 × L × E components) can be represented as y = Dx, where D is the matrix that defines the over-complete dictionary whose columns are called atoms and x is the sparse representation of y. This means that only a reduced number of columns of D are linearly combined to approximate the vector x.

Dictionaries in the Sparse Representation Classifiers (SRC) are usually composed by arranging training samples from the different classes in columns in a matrix. However, there are dictionary computation algorithms, such as K-SVD (Singular Value Decomposition approach to K-means) [18], which allow the selection of dictionary atoms that maximize the representative capabilities of the dictionary. The dictionary D can be learnt from training samples by minimizing the reconstruction error while complying with the *sparsity* constraint. In this work, we use the K-SVD algorithm due to its representation capabilities. The classifier shown in Fig. 4 is based on this *bag of words* paradigm also described in [19]. This classification model allows discriminative features to be extracted from the data manifold, and has provided good results for time series classification. However, the model we used in this work tries to leverage the classification capabilities by using a bag of sparse features and either a multiclass linear Support Vector Classifier (SVC) or a LDA classifier. Specifically, the proposed classification paradigm is composed of three stages. Once the coefficients of the considered MRA have been computed from EEG training data, a sparse dictionary is computed independently for each class using the K-SVD algorithm [18]. Since the dictionary computed by K-SVD maximizes the representation of the samples but not in a discriminative way, we compute a different dictionary for each class, and then all the dictionaries are concatenated to form a unique dictionary. Subsequently, the sparse representation of each training sample is computed by means of the dictionary using the Orthogonal Matching Pursuit (OMP) algorithm [20]. These sparse features computed for each segment are summed up for all the segments in each trial, representing that trial by a histogram of sparse features. Finally, histogram codewords (in our case, the sum of the coefficients) are used to train either a linear multiclass SVC or a LDA classifier following the one-against-all strategy. The testing process uses the dictionary and the codebook computed during the training phase to calculate the histogram codewords as well to predict the class of a test sample using the previously trained multiclass SVC or LDA classifier.

### Three approaches for multiobjective feature selection

**Fig. 5:**
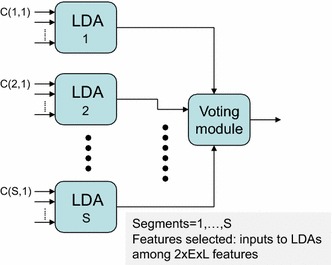
EEG classification with multiple LDA classifiers based on majority voting, with one LDA classifier per segment

In this paper we propose three feature selection approaches for the MRA in the classification of BCI imagery tasks. The first alternative simply searches a subset of features (inputs) among the whole set of 2 × S × E × L features as the input to an LDA. The second alternative takes into account the structure of Fig. 3, where it is clear that it is possible to select among the LDAs as the input to the voting module. Finally, the third proposed approach implements the structured LDAs as shown in Fig. 5. In this case, there is one LDA per segment, and it is necessary to select the input to each LDA. While the feature searching space in the classifier structure of Fig. 3 has a dimension of 2 × S × L, the classifiers in Fig. 5 corresponding to the third proposed alternative have an input dimension of 2 × E × L. More details about these approaches will be given in the next section.

## Results

This section presents the experimental results obtained by the evolutionary multiobjective feature selection approaches, in comparison with the baseline methods described in the previous section. The experiments have been performed by using the dataset recorded in the BCI Laboratory at the University of Essex. For each subject, there is one data file named x1## with data recorded in two runs for training and another data file named xe1## with data recorded in two runs for evaluation. Each data file contains about 180 labelled patterns with data from 20 segments (S = 20), six levels (L = 6) of approximation or detail coefficients (a/d = 2), and 15 electrodes (E = 15). The class labels correspond to three imagined movements of right hand, left hand, and feet.

One of the baseline methods for comparison (OPT0) corresponds to the MRA framework depicted in Fig. 3, where all the possible S × L × 2 LDAs are considered for voting the class to which the corresponding pattern belongs. The number of inputs to each LDA is the same as the number of electrodes. The input from a given electrode is the normalized second moment of the wavelet coefficients of the signal in this electrode for the s-th segment, the l-th level, and approximation/detail type of the corresponding LDA. In OPT0 there is no feature selection. As shown in Fig. 4, the other two baseline methods SR-LDA and SR-SVC correspond to the approaches based on the sparse representation. In these sparse representation methods we have used a dictionary of 30 atoms per class (i.e., a dictionary of 90 atoms after concatenating the atoms per class is obtained). To generate these dictionaries with K-SVD, combinations of seven elements per linear combination have been used. We have considered that at most 15 coefficients are different from zero in the OMP algorithm. This sparsity constraint of 15 has been set as it provides the best results after some experiments.

The three methods proposed in this paper (OPT1, OPT2, and OPT3) use different multiobjective feature selection approaches. OPT1 uses one LDA classifier only, with the multiobjective feature selection procedure applied on all the S × L × E × 2 features corresponding to the possible segments, levels, variances of the approximation and detail coefficients, and electrodes. With the BCI dataset of the University of Essex there are 20 × 6 × 15 × 2 = 3600 possible features for selection for OPT1. Similar to OPT0, OPT2 and OPT3 also implement a structure that uses a voting module to determine the output of the classifier from the most frequent output of the LDAs. OPT2 is based on the classifier structure shown in Fig. 3. In this case, the feature selection problem is to select among the S × L × 2 possible LDAs used for voting in the OPT0 method. This means that the dimension of the search space is 20 × 6 × 2 = 240. OPT3 is based on the classification scheme shown in Fig. 5. In this case, the number of LDAs used for voting is equal to the number of segments, and each LDA can have up to 2 × E × L features as inputs. With the BCI dataset, there are 20 LDAs, each with up to 2 × 6 × 15 = 180 inputs. There are 2 × S × L × E features, but they are structured and no more than 2 × E × L features are available for selection for a given LDA. As the number of patterns is less than 180 in both the training and testing sets, only less than 30 features are selected by the multiobjective evolutionary feature selection algorithm.

The evolutionary multiobjective feature selection procedures have been executed with populations of 20, 30, and 50 individuals respectively, and with 20, 30, and 50 generations respectively to determine the minimum number of individuals and generations that provide competitive results compared with OPT0. OPT1, OPT2, and OPT3 have been executed by using 50 individuals and 50 generations so that the amount of searching work in all the approaches is similar. Executions with different number of iterations and individuals would provide a fairer comparison among the different approaches. We will consider this at the end of this section.

As has been said, the multiobjective optimization algorithm implemented in our wrapper procedures is NSGA-II [15]. As evolutionary operators, we have used simulated binary crossover with a crossover probability of 0.5, a mutation probability of 0.5, and distribution index of 20 for crossover and mutation operators as these parameters have provided competitive results in our experiments. Although the mutation probability could be considered high, it seems that the elite-preserving mechanism implemented in NSGA-II has preserved enough non-dominated solutions to build adequate Pareto-front approximations. It is worth mentioning that no work on tuning the parameters of the evolutionary multiobjective feature selection options to optimize their behavior has been considered, as our aim here is to analyze whether multiobjective optimization is able to provide some improvements on MRA approaches for BCI.

With respect to the two cost functions used in the multiobjective algorithm, C1 is related to the Kappa index [16] (C1 = 1 − Kappa index) obtained after the learning iterations executed in the feature evaluation step of the evolutionary algorithm. The Kappa index takes into account the distribution of the per class error as it is computed as (p_0_ − p_c_)/(1 − p_c_) with p_0_ equal to the proportion of coincidences among the classification outputs and the labels of the patterns and pc being the proportion of patterns on which the coincidence is expected by chance.

In this feature evaluation step, the cost function C2 is computed as the average loss function in a tenfold cross validation analysis to the training patterns. Thus, to compute C2, the set of training patterns are partitioned into 10 equal sized subsets and the cross-validation process is repeated 10 times, by using one of the 10 training subsets as validation data for testing the classifier, and the remaining patterns as training patterns to determine the classifier. The mean squared classification error over the 10 cross-validation processes is precisely the value of C2. This way, all the available training patterns are considered for both training and testing, with each pattern being used for testing once. The use of k-fold cross validation to evaluate the possible overfitting is also considered in [21] where this approach is also considered in relation with most traditional generalization methods based on pruning techniques.

The relation between these two cost functions, C1 and C2, is illustrated in Table 1, which shows the correlations between C1 and C2 in the populations obtained, in 15 executions of the multiobjective feature selection algorithm, for subjects 104 and 107. As it can be seen, except in one case, statistically significant correlations are negative. In the case with statistically significant positive correlation, the correlation is small.

**Table 1:** Correlations between C1 and C2 for subjects 104 and 107 for 15 executions of the multiobjective selection algorithm OPT1

Rep.	x104		x107	
	Corr(C1,C2)	p	Corr(C1,C2)	p
1	−0.8540	0.15e−28	−0.7870	0.29e−21
2	−0.9652	0.66e−58	0.0	0.0
3	−0.0594	0.55	−0.3282	0.86e−3
4	−0.9007	0.29–36	−0.8899	0.35e−34
5	−0.7492	0.31e−18	−0.8652	0.39e−30
6	0.0	0.0	−0.8986	0.77e−36
7	0.2619	0.0085	−0.8897	0.38e−34
8	−0.6880	0.26e−14	−0.7870	0.28e−21
9	0.0421	0.6778	0.0	0.0
10	−0.6509	0.23e−12	−0.3282	0.86e−3
11	−0.9199	0.12e−40	−0.8899	0.35e−34
12	0.1861	0.0637	−0.8652	0.39e−30
13	−0.9899	0.53e−84	−0.8986	0.77e−36
14	−0.9589	0.21e−54	−0.8897	0.38e−34
15	−0.2821	0.0045	−0.1685	0.09

**Fig. 6:**
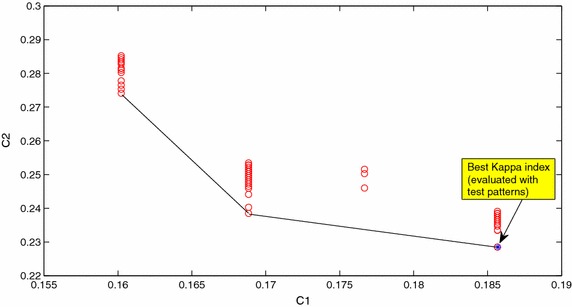
Cost functions C1 and C2 after an execution of OPT1 for subject 107

Figure 6 shows the values of C1 and C2 for a population of individuals obtained by the multiobjective feature selection procedure OPT1 for subject 107. As can be seen, one of the non-dominated solutions (in the obtained Pareto front approximation) is one of the best solutions once the Kappa indices of the population have been evaluated by using the test patterns. It can be seen that this best solution is not included in the set of solution with the lowest value obtained by the multiobjective feature selection algorithm for C1.

Table 2 compares the Kappa indices obtained by the different approaches, OPT0 to OPT3, and SR-LDA based on LDA and SR-SVC based on SVC. The columns labeled as “Kappa index (xe#)” provide the Kappa index values obtained by the 6 approaches when the testing patterns were used to evaluate the classifier performance for each subject. The results shown in Table 2 for SR-LDA, SR-SVC, OPT1, OPT2, and OPT3 are the average (mean and standard deviation) over 15 executions of each approach for each subject. The statistical analysis has been conducted by applying a Kolmogorov–Smirnov test first to determine whether the obtained values of the Kappa index follow a normal distribution or not. If the experimental results do not have normal distribution, a non-parametric Kruskal–Wallis test has been used to compare the means of the different algorithms.

**Table 2:** Comparison of different feature selection and classification methods for the University of Essex BCI data files (Kappa values evaluated with the test patterns)

Subject	OPT0	SR-LDA	SR-SVC	OPT1	OPT2	OPT3
	Kappa index (xe#)	Kappa index (xe# mean,std)	Kappa index (xe# mean,std)	Kappa index (xe# mean,std)	Kappa index (xe# mean,std)	Kappa index (xe# mean,std)
101	0.438	0.365 ± 0.045	*0.444* *±* *0.048*	0.393 ± 0.046	0.437 ± 0.033	0.367 ± 0.032
102	*0.455*	0.347 ± 0.050	0.417 ± 0.031	0.302 ± 0.074	0.429 ± 0.023	0.382 ± 0.044
103	0.279	0.186 ± 0.047	0.196 ± 0.058	0.249 ± 0.046	0.325 ± 0.017	*0.356* *±* *0.024*
104	0.564	0.607 ± 0.057	*0.614* *±* *0.043*	0.510 ± 0.056	0.545 ± 0.035	0.563 ± 0.034
105	*0.287*	0.110 ± 0.035	0.105 ± 0.045	0.191 ± 0.040	0.240 ± 0.031	0.227 ± 0.023
106	*0.321*	0.160 ± 0.044	0.181 ± 0.034	0.193 ± 0.070	0.319 ± 0.028	0.246 ± 0.036
107	0.631	0.464 ± 0.048	0.507 ± 0.046	0.560 ± 0.041	*0.634* *±* *0.019*	0.603 ± 0.027
108	*0.254*	0.095 ± 0.056	0.101 ± 0.062	0.088 ± 0.036	0.184 ± 0.027	0.184 ± 0.028
109	*0.388*	0.228 ± 0.025	0.262 ± 0.043	0.207 ± 0.071	0.333 ± 0.026	0.321 ± 0.037
110	*0.648*	0.443 ± 0.056	0.456 ± 0.069	0.450 ± 0.036	0.605 ± 0.041	0.578 ± 0.027

Italic values represent the best values provided by any alternative procedure for a given subject

**Fig. 7:**
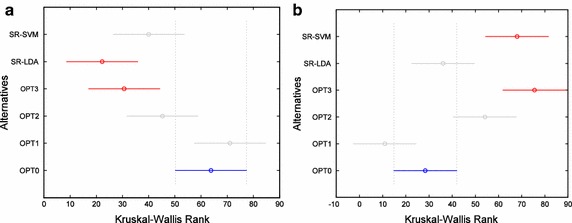
Multiple comparison test for subjects 104 (**a**) and 107 (**b**). *Blue* *interval* indicates OPT0. *Red intervals* (SR-LDA and OPT3 in **a** and SR-SVM and OPT3 in **b**) indicate alternatives that provide results different from OPT0 at a significance level of 5 %

Table 3 shows the p values of the statistical tests of differences in performances of feature selection approaches for each subject with a significance level of 5 % (the probability for rejecting the null hypothesis when it is true is below 0.05). Moreover, multiple comparison tests have been performed to identify which alternatives are different, with a significance level of 5 %, in those subjects providing the best Kappa values. Thus, a deeper analysis can be made taking into account the confidence intervals obtained from the multiple comparison tests in order to identify those pairs showing differences at the significance level of 5 %. Table 4 lists the group of best performing procedures for each subject. Figure 7 shows some results of the multiple comparison tests implemented through Kruskal–Wallis test for subjects 104 (Fig. 7a) and 107 (Fig. 7b), which provides the intervals of the Kruskal–Wallis rank for each considered alternative (OPT0 to OPT1, SR-LDA, and SR-SVD). From Fig. 7 it is clear that OPT3 and SR-LDA for subject 104 and OPT3 and SR-SVC for subject 107 provide the most different results with respect to OPT0, with a significance level of 5 %.

**Table 3:** Results of the Kruskal–Wallis test (p values below 0.05 mean statistically significant differences)

Subject	*p* value
101	1.251e−06
102	8.804e−12
103	2.362e−14
104	2.014e−07
105	4.286e−13
106	2.705e−12
107	3.238e−13
108	6.005e−15
109	1.186e−12
110	1.860e−14

**Table 4:** Best performing procedures for each subject (differences with a significance level of 5 %)

Subject	Group of best procedures
101	SR-SVM, OPT0, OPT2
102	SR-SVM, OPT0, SR-LDA
103	OPT1, OPT0, SR-SVC
104	OPT2, OPT3, SR-LDA
105	OPT0, OPT2, OPT3
106	OPT0, OPT1, SR-LDA
107	OPT0, OPT1, OPT3
108	OPT0, OPT1, SR-LDA
109	OPT0, SR-SVC, OPT1
110	OPT0, SR-LDA, OPT1

Table 5 shows the best values of the Kappa index achieved by each approach over 15 executions. Compared to OPT0, SR-LDA and SR-SVC, feature selection (by OPT1, OPT2, or OPT3) is able to provide competitive results, with the advantage that fewer features are required. In some cases, OPT2 provides even better classification performance than OPT0. According to the statistical tests, except for subjects 108, 109, and 110, OPT2 is able to obtain the same or even better results than OPT0, with fewer features (less than thirty features). This is highly valuable when designing online BCI systems.

**Table 5:** Comparison of different feature selection and classification methods for the University of Essex BCI data files: maxima Kappa values evaluated with the test patterns

Subject	OPT0	SR-LDA	SR-SVC	OPT1	OPT2	OPT3
	Kappa index (xe#)	Kappa index (xe# max)	Kappa index (xe# max)	Kappa index (xe# max)	Kappa index (xe# max)	Kappa index (xe# max)
101	0.438	0.450	*0.528*	0.472	0.489	0.430
102	0.455	0.408	*0.541*	0.405	0.463	0.447
103	0.279	0.244	0.287	0.329	0.354	*0.413*
104	0.564	*0.696*	0.664	0.589	0.614	0.606
105	*0.287*	0.194	0.170	*0.287*	*0.287*	*0.287*
106	0.321	0.236	0.284	0.338	*0.381*	0.292
107	0.631	0.547	0.598	0.631	*0.665*	0.656
108	*0.254*	0.161	0.155	0.170	0.245	0.237
109	*0.388*	0.320	0.380	0.346	0.371	*0.388*
110	0.648	0.505	0.530	0.530	*0.673*	0.639

Italic values represent the best values provided by any alternative procedure for a given subject

With respect to the execution time required by each approach, the mean execution time is 4533 ± 45 s for OPT1, 13,353 ± 1031 s for OPT2, and 1159 ± 56 s for OPT3. Taking into account these differences in the running time, it is possible to argue that the comparison among different approaches may not be fair as OPT1 and OPT3 could probably achieve better results when more generations (with more individuals) are adopted with execution time similar to that required by OPT2. Table 6 shows the results obtained, for two subjects only (×104 and ×107), with OPT1 using a population of 100 individuals and 60 generations and OPT3 using 200 individuals and 90 generations. When 100 individuals and 60 generations are used, OPT1 requires a mean execution time of 11,042 ± 162 s, which is in a similar order as the one required by OPT2. In the case of OPT3, 200 individuals and 90 generations still consume less execution time (8408 ± 144 s) than OPT2 with 50 individuals and 50 generations.

**Table 6:** Comparison of Kappa indices for OPT1 with 100 individuals and 60 generations, and OPT3 with 200 individuals and 90 generations, with respect to OPT1, OPT2, and OPT3 with 50 individuals and 50 generations

Subject	OPT1 (100,60)	OPT1 (50,50)	OPT2 (50, 50)	OPT3 (200,90)	OPT3 (50,50)
	Kappa index (x# mean, std)	Kappa index (x# mean, std)	Kappa index (x# mean, std)	Kappa index (x# mean, std)	Kappa index (x# mean, std)
104	0.841 ± 0.018	0.819 ± 0.013	*0.902* *±* *0.010*	0.897 ± 0.019	0.882 ± 0.022
107	0.836 ± 0.016	0.816 ± 0.018	*0.880* *±* *0.011*	0.865 ± 0.018	0.845 ± 0.022

Subject	OPT1 (100,60)	OPT1 (50,50)	OPT2 (50, 50)	OPT3 (200,90)	OPT3 (50,50)
	Kappa index (xe# mean, std)	Kappa index (xe# mean, std)	Kappa index (xe# mean, std)	Kappa index (xe# mean, std)	Kappa index (xe# mean, std)
104	0.515 ± 0.047	0.510 ± 0.056	0.545 ± 0.035	*0.573* *±* *0.032*	0.563 ± 0.034
107	0.580 ± 0.052	0.560 ± 0.041	0.634 ± 0.019	*0.644* *±* *0.022*	0.603 ± 0.027

*x#* evaluation was done with training patterns, *xe#* evaluation was done with test patterns

Italic values represent the best values provided by any alternative procedure for a given subject

The results in Table 6 show improvements on the performance of OPT1 and OPT3 as the number of individuals in the population and generations are increased. In the values of the Kappa index obtained by using the training patterns (x# columns), the Kruskal–Wallis test shows that the differences are statistically significant for OPT1 (p = 0.00 and 0.005 for ×104 and ×107, respectively). However, when the evaluation was done by using the test patterns (xe# columns), these differences among OPT1 with 100 and 50 individuals are not significant (p = 0.75 and p = 0.25 for 104 and 107, respectively). In the case of OPT3, the situation is similar for the values of Kappa index obtained by using the training patterns (p = 0.04 and 0.014 for 104 and 107, respectively). With respect to the values of Kappa index obtained by using the testing patterns, the results are not statistically significant for 104 (p = 0.63) but are statistically significant for 107 (p = 0.005). It can be seen that OPT3 with a population of 200 individuals and 90 iterations achieved better performance than OPT2 (evaluation with the test patterns, i.e., xe# columns). Nevertheless, the Kruskal–Wallis test only shows statistical significance for 107 (p = 0.005 for 107, and p = 0.0997 for 104).

## Discussion and conclusion

Procedures such as the one described in [8] provide approaches to cope with the curse of dimensionality based on the composition of multiple classifiers. Each classifier receives only a subset of pattern components (features) in such a way that the number of training patterns is much higher than the number of features used as inputs. The problem with these approaches is that the number of classifiers to train and to accomplish the classification is usually very high (as all the features should be taken into account). Furthermore these approaches do not provide information about the most relevant features, and the need to compute such a large number of features and to train a lot of classifiers could be a significant drawback to satisfy real-time requirements of many applications. In this context, the contribution of this paper is twofold. We have proposed a multiobjective approach to cope with the feature selection in LDA classification based on two cost functions that evaluate the classifier’s accuracy and its generalization capability. On the other hand, we have proposed several structures for the classifier to take advantage of our multiobjective approach to feature selection in different ways (OPT1–OPT3). These proposals have been compared with the approach based on the composition of multiple classifiers without using feature selection (OPT0), and with two alternatives based on sparse representation for feature definition that respectively use LDA (SR-LDA) and SVC (SR-SVC) as classifiers.

The experimental results show that evolutionary multiobjective feature selection is able to provide classification performance similar to that of using all the possible LDAs with all the possible feature inputs (OPT0) and the other two alternatives based on sparse representation of features (SR-LDA and SR-SVC). However, the proposed approaches lead to simpler classification procedures with fewer features. As a matter of fact, the classification performances obtained by OPT1 to OPT3 correspond to solutions with less than 30 features.

Multiobjective optimization has been applied for feature selection inside a wrapper method in a multiresolution analysis (MRA) system for BCI applications. The comparison with other alternatives, including different classifiers, structures of classifiers, and approaches to define the features (for example by sparse representations), shows competitive performances of the multiobjective approaches with fewer features than the other considered alternatives. The performance observed is quite different among subjects, but all the procedures behave homogeneously better or worse in a similar way for each subject.

Besides the analysis of the characteristics of the selected features for obtaining some knowledge about important electrodes and segments, etc., there are other issues that can be considered to improve the performance of multiobjective feature selection in BCI applications. It is clear that improving the cost function that evaluates the generalization capability is an important issue to study, along with the evaluation of multiobjective feature selection by using different advanced classifiers, such as SVM, or even the comparison of our approach with deep learning, which can be considered as a powerful framework to cope with data of high-dimensionality [22, 23]. Moreover, instead of using the normalized variance as a first approach to reduce the number of coefficients as in [8], the use of entropy could provide some advantages in the case of wavelet coefficients [24]. Finally, the implementation of cooperative coevolutionary approaches able to cope with problems with a large number of decision variables (features) is also an interesting topic [25].
